# Control of leaf development in the water fern *Ceratopteris richardii* by the auxin efflux transporter *CrPINMa* in the CRISPR/Cas9 analysis

**DOI:** 10.1186/s12870-024-05009-4

**Published:** 2024-04-24

**Authors:** De-Liang Xiang, Gui-Sheng Li

**Affiliations:** College of Biological Resources and Environmental Sciences, Jishou University, Jishou, 416000 China

**Keywords:** *PIN*, Leaf development, Ferns, Gene duplication, Genome editing

## Abstract

**Background:**

*PIN-FORMED* genes (*PIN*s) are crucial in plant development as they determine the directionality of auxin flow. They are present in almost all land plants and even in green algae. However, their role in fern development has not yet been determined. This study aims to investigate the function of *CrPINMa* in the quasi-model water fern *Ceratopteris richardii*.

**Results:**

*CrPINMa* possessed a long central hydrophilic loop and characteristic motifs within it, which indicated that it belonged to the canonical rather than the non-canonical *PIN*s. *CrPINMa* was positioned in the lineage leading to *Arabidopsis PIN6* but not that to its *PIN1*, and it had undergone numerous gene duplications. CRISPR/Cas9 genome editing had been performed in ferns for the first time, producing diverse mutations including local frameshifts for *CrPINMa*. Plants possessing disrupted *CrPINMa* exhibited retarded leaf emergence and reduced leaf size though they could survive and reproduce at the same time. *CrPINMa* transcripts were distributed in the shoot apical meristem, leaf primordia and their vasculature. Finally, *CrPINMa* proteins were localized to the plasma membrane rather than other cell parts.

**Conclusions:**

CRISPR/Cas9 genome editing is feasible in ferns, and that *PIN*s can play a role in fern leaf development.

**Supplementary Information:**

The online version contains supplementary material available at 10.1186/s12870-024-05009-4.

## Background

Auxin phytohormones are ubiquitous in the plant kingdom, and polar auxin transport (PAT) can impact lots of developmental events. There are several influx and efflux auxin transporters, and among them PIN-FORMED transporters (PINs) determine the direction of auxin flow within tissues [1]. PINs have both a N-terminal and a C-terminal transmembrane region. Moreover, PINs possessing a long central hydrophilic loop located between the two transmembrane regions are typically localized to the plasma membrane (PM), which is crucial to auxin transportation from the intracellular to the extracellular space. In contrast, PINs bestowed a short hydrophilic loop are generally localized to the endoplasmic reticulum (ER) and thus answer for intracellular auxin homeostasis. PINs act as homodimers and can bind indole-3-acetic acid (IAA), or even N-(1-naphthyl)phthalamic acid (NPA) at the same site but with a higher affinity [2, 3]. PINs transport auxin through the PM like elevators [4].

Newly synthesized PINs are evenly allocated to the PM. However, after endocytosis, recycling, or degradation, PINs will become unevenly distributed throughout the PM. One significant reason for this polar distribution is phosphorylation/dephosphorylation [5–7]. PINs contain a series of conserved sites that can be acted upon by kinases/phosphatases, which are nested in the long central hydrophilic loop residing at the cytoplasmic side and can be utilized in different combinations [8, 9]. As a result, overexpression of some kinases has induced the basal-to-apical re-localization of PINs in embryos and roots, while kinase loss-of-function can cause the apical-to-basal re-polarization of PINs in inflorescence apexes [10]. Certainly, phosphatases may counteract these shifts [11]. Finally, auxin itself can regulate the polar localization of PINs in the PM at both transcriptional and post-translational levels [12, 13].

Leaf venation is fascinating and leaf vasculature forms due to the differentiation of the procambial cells that arise from the subepidermal ground meristem. Before the formation of the procambial cells, *PIN*s have been expressed and their protein products are always localized to the cell side that faces the pre-existing vasculature. The PM polarity of PINs will change in leaf epidermal cells, which is necessary for vein initiation. At the same time, the ground meristem cells may become bipolar in terms of PIN distribution for vein connection, whereas intercalating cells can acquire a PIN distribution polarity for vein extension [14, 15]. Leaves can be classified as either simple, namely possessing a single continuous blade, or compound, namely consisting of multiple leaflets. In compound leaves, leaflet initiation can be predicted based on PIN convergence [16]. Loss-of-function of *PIN*s may result in simple rather than compound leaves [17]. *PIN*s are frequently expressed at the sites of future leaf primordia, thus affecting the formation of phyllotaxis and plastochron (the frequency of leaf initiation) [18, 19]. Finally, *PIN*s can play a role in leaf flattening [20], leaf serration [21], and interdigitation of leaf pavement cells [22].

Both streptophytes and chlorophyta possess *PIN*s, despite they diverged from each other 1.2 billion years ago [23]. The PAT has also been observed in both algae and mosses [24, 25]. In ferns, *PIN*s can function for a rudimentary root gravitropism [26]. However, it is unknown for the contribution of *PIN*s to fern leaf development. *Ceratopteris richardii* is a quasi-model species in ferns, making it appealing to perform genome editing to investigate functions of its *PIN*s. In the bacterial type II clustered regularly interspaced short palindromic repeat (CRISPR) and CRISPR/associated (Cas) protein system, a guide RNA (gRNA) can be designed to target the genomic 5’-N20-NGG-3’ (N indicating any base), where N20 corresponds to the so-called spacer and NGG represents the protospacer-adjacent motif (PAM) [27–29]. Multiple gRNAs can be generated from a single DNA construct through exploiting the endogenous RNases that can regularly cleave tRNA molecules for their maturation [30]. Genome editing can bring about various outcomes, such as heterozygous mutation for one allele, homozygous mutation for two alleles, biallelic mutation that possesses different alterations for each allele, and chimeric mutation concerning multiple cells [31].

*CrPINMa* was found in sequences like canonical *PIN*s and had undergone intensive gene duplications. Its null mutation could be generated with the CRISPR/Cas9 genome editing, and the resultant loss-of-function could retard leaf initiation and reduce leaf size. *CrPINMa* could be expressed in leaf vasculature and its proteins could be localized to the PM. Therefore, *PIN*s also control leaf development in ferns.

## Results

### Phylogeny of *CrPINMa*

**Fig. 1:**
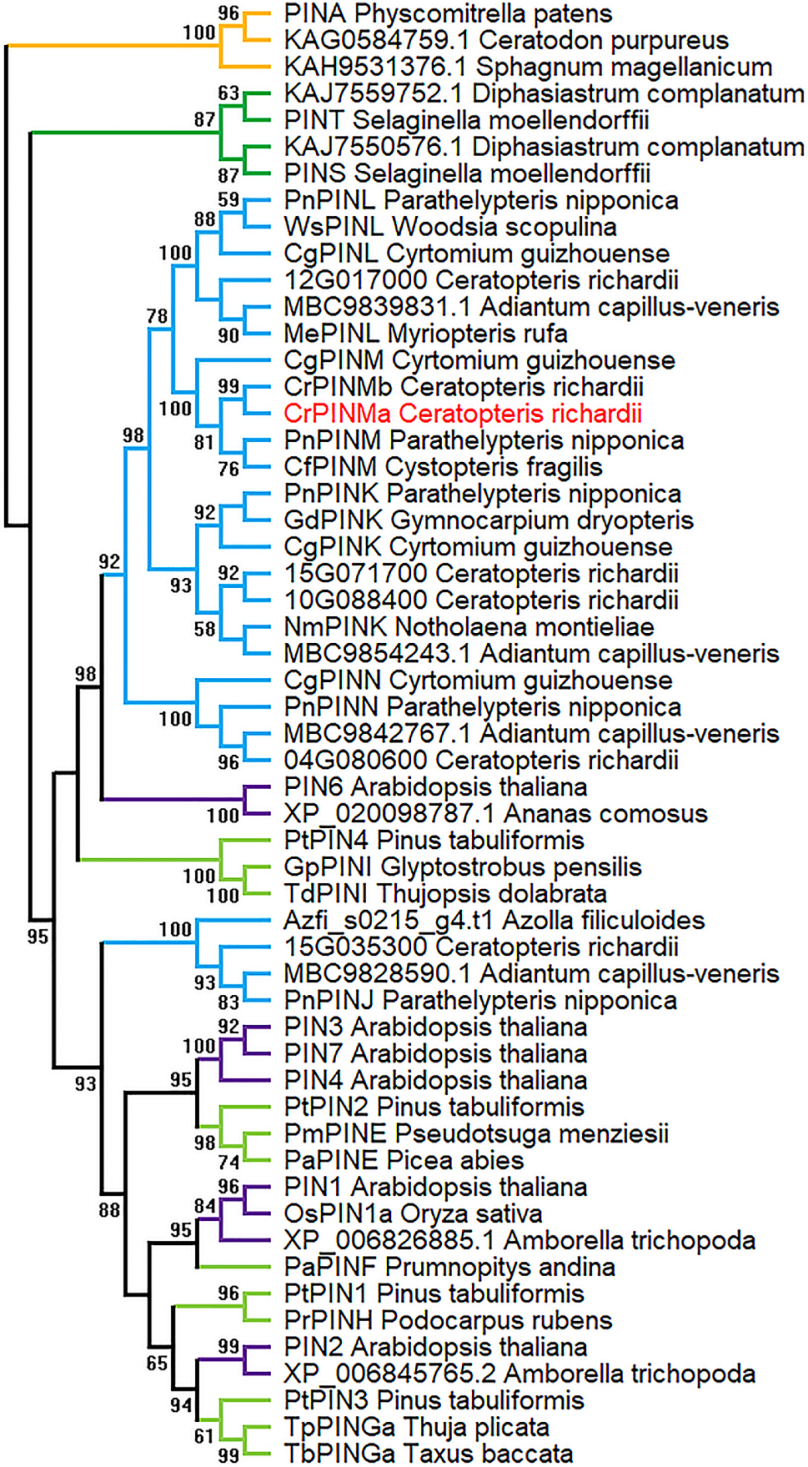
A phylogenetic tree of representative *PIN*s. Numbers along branches were bootstrap values (hidden if < 50%). Gene names/accessions and species names were shown at every terminal branch. Branches were colored to indicate different land plants, e.g., bryophytes in gold, lycophytes in green, ferns in blue, gymnosperms in light green, and angiosperms in purple. The investigated *CrPINMa* was highlighted by the name in red

*CrPINMa* was located at the chromosome locus 29G075900 and had a 2025 bp (bp) coding sequence. This coding sequence concerned six exons and answered for a protein with five transmembrane regions in both the N- and the C-terminus [see Fig. S1]. *CrPINMa* belonged to the PINM clade (100% supported) sister to the PINL clade (100%) (Fig. 1). *CrPINMa* was initially clustered with another gene from the same species, which indicated a recent gene duplication. *CrPINMa* was further clustered with genes from the PINK (93%) and the PINN (100%) clades, thus be related to three gene duplications that occurred in the common ancestor of the involved ferns, roughly the core leptosporangiates. Additionally, *CrPINMa* was found to be part of a larger clade (98%) that had encompassed *PIN6* from *Arabidopsis thaliana*. *CrPINMa* was even in the same clade with gymnosperm PINI genes, albeit under a support below 50%. *CrPINMa* was different from remaining fern *PIN*s since the latter were clustered with other angiosperm and gymnosperm genes (93%). Therefore, *CrPINMa* was also associated with a gene duplication that occurred in the common ancestor of ferns and seed plants. In the tree that had included more divergent genes, *CrPINMa* was still positioned in such a manner except for the relationship with gymnosperm PINI genes (see Fig. S2).

*CrPINMa* had a long central hydrophilic loop (approximately 362 amino acids) (see Fig. S2). This loop was longer than that of *Arabidopsis PIN1* (approximately 309 amino acids) and ten times longer than the short central hydrophilic loop of *PIN5* (approximately 36 amino acids). *CrPINMa* contained motifs that were common to most *PIN*s, which, however, were absent from algal homologs and *PIN*s with a short central hydrophilic loop, such as *PIND* from *Physcomitrella patens*. *CrPINMa* contained four Motif 2 arranged in tandem, and their arrangement was the same in other PINM genes and all PINK genes. However, PINL genes contained only three Motif 2, whereas PINN genes contained just two such motifs. The more distant PINI genes sometimes contained only one Motif 2. In extrapolation, PIN6 lacked Motif 2 and only contained Motif 1 and Motif 7.

### Mutations from genome editing

**Fig. 2:**
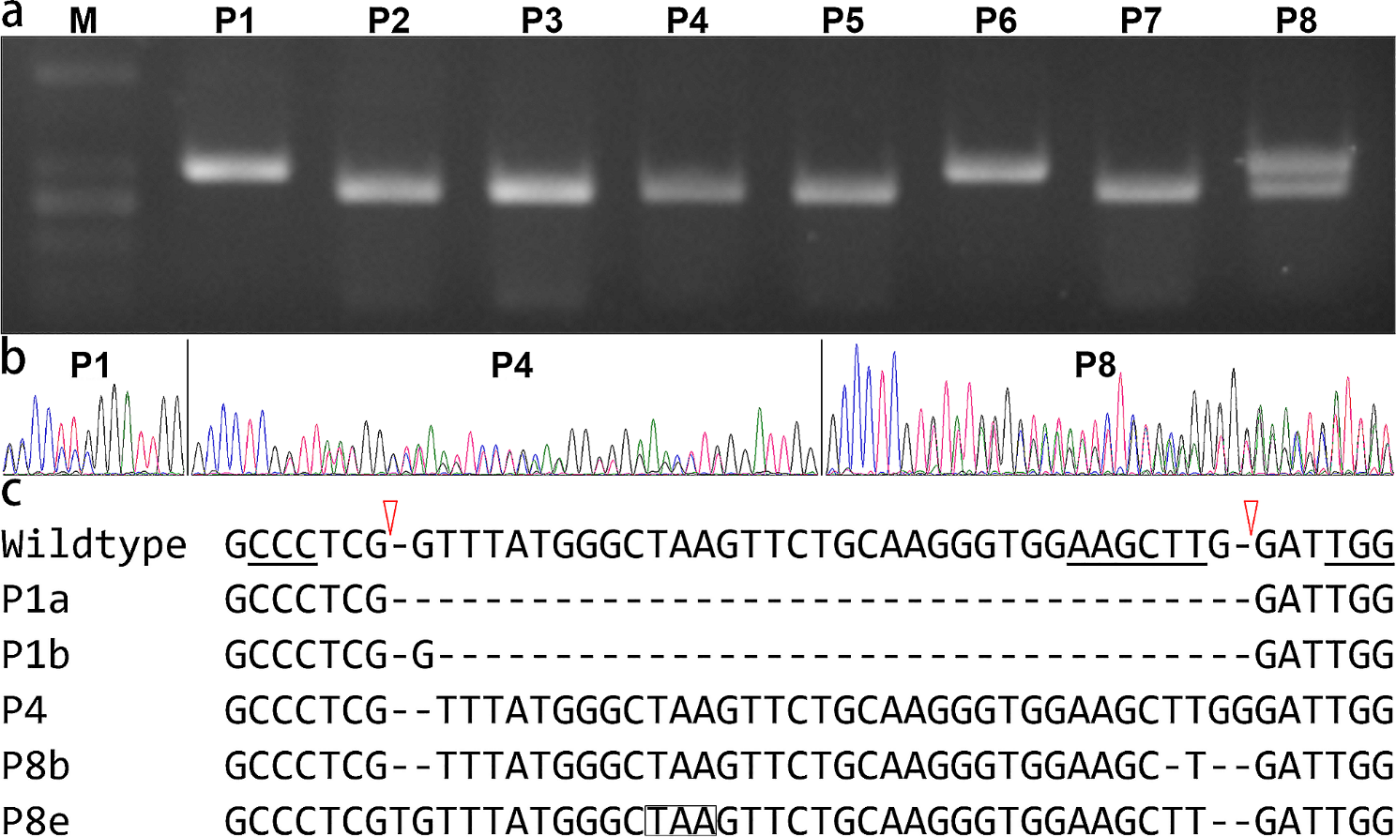
Identification of the edited plants. (**a**) Electrophoresis of the HindIII-digested PCR products spanning the two gRNA binding sites. M, molecular marker. P1–P8, investigated individuals. (**b**) Sequencing of the same PCR products. Double peaks suggested that there were two different alleles. (**c**) Mutation characterization of the edited alleles. The wildtype indicated an in-frame coding region spanning the two PAM motifs, which were highlighted by underlines. The HindIII site was also underlined, and the cutting positions by the editing machine were indicated by two red triangles. The P8e allele contained a premature stop codon highlighted by boxing

Eight plants were investigated, and genome editing was observed in individuals P1, P4, P6, and P8. Enzymatic cutting analysis showed that P1 and P6 performed like homozygous plants in terms of *CrPINMa* (Fig. 2a). However, sequencing revealed that P1 possessed two different mutated alleles (Fig. 2b), with P1a harboring a 35-bp deletion and P1b a 34-bp deletion (Fig. 2c). These deletions removed the diagnostic HindIII site. Furthermore, the P1 progenies were sequenced, resulting in the discovery of four P1a homozygotes, one P1b homozygote, and two *P1a P1b* heterozygotes. Thus, the wild-type allele had not been observed in the P1 line. Clone sequencing confirmed that P6 was identical to P1. As anticipated, genome editing occurred after the third nucleotide proceeding the PAM motif, and it had happened to both gRNAs. Consequently, out-of-frame mutation had occurred to *PINMa* in P1/P6. P4 performed like wild-type plants in the digestion assay, despite having one wild-type allele and one allele with one T-deletion in one gRNA site and one G-insertion in the other site. Nevertheless, this affected allele retained the HindIII site. Therefore, P4 was compromised in *CrPINMa*, with one allele experiencing a local frameshift restricted to the region between the pair of gRNA sites, but free of any premature stop codons. P8 possessed *CrPINMa* alleles that were either resistant or sensitive to HindIII digestion. The P8e allele had one T-insertion in one gRNA site and one G-deletion in the other site, resulting in a local frameshift and a nested premature stop codon. The P8b allele had one G-deletion in one gRNA site and one T-deletion in addition to one G-deletion (or one TG-deletion) in the other site, resulting in a local frameshift without premature stop codons.

### Maintenance of mutations in transcripts

**Fig. 3:**
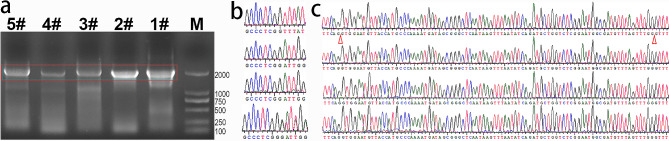
*CrPINMa* transcript analysis in edited plants. (**a**) Electrophoresis of RT-PCR products. The DNA ladder (M), a wild-type plant (1#), and four P1 progeny (2#, 3#, 4#, 5#) were included and the expected bands were boxed. (**b**) Sequencing of RT-PCR products focusing on the edited site. The top one (1#) showed the original sequence, the underneath one (2#) and the third one (4#) displayed the same mutation, namely the P1a mutation. The bottom one (5#) displayed the P1a and P1b transcripts. (**c**) Sequencing focusing on the intron region. The same sequence was generated in 1#, 2#, 4# and 5# (from top to bottom). Two red triangles indicated intron positions

The P1 line was chosen to clarify the maintenance of mutations in gene transcripts because it was mutated in both *CrPINMa* alleles, and the two mutations were loss-of-function, useful for implicating gene function. *CrPINMa* was still expressed in the progeny of P1, as the specific electrophoresis bands were generated from RT-PCR, and they roughly indicated a length of 1924 bp, which was expected based on *CrPINMa* transcripts, whereas the corresponding genomic sequence was at least of 3200 bp (Fig. 3a). One sample (4#) produced a smaller band, because a shorter sequence had been generated in RT-PCR. *CrPINMa* was also mutated regarding transcripts in the progeny of P1, displaying P1a mutation or P1a and P1b mutations simultaneously (Fig. 3b). *CrPINMa* transcripts had skipped a region of 97 nt in one sample (4#), seemingly due to an assumed GU-AG intron boundary. The RT-PCR products had not been fully sequenced, and therefore *CrPINMa* possibly had generated more abnormal transcripts in the progeny of P1. These were transcripts that were examined because none of the introns had been sequenced, which for *CrPINMa* would normally be the second and third introns in genomic DNA sequencing (Fig. 3c).

### Morphologies of edited plants

**Fig. 4:**
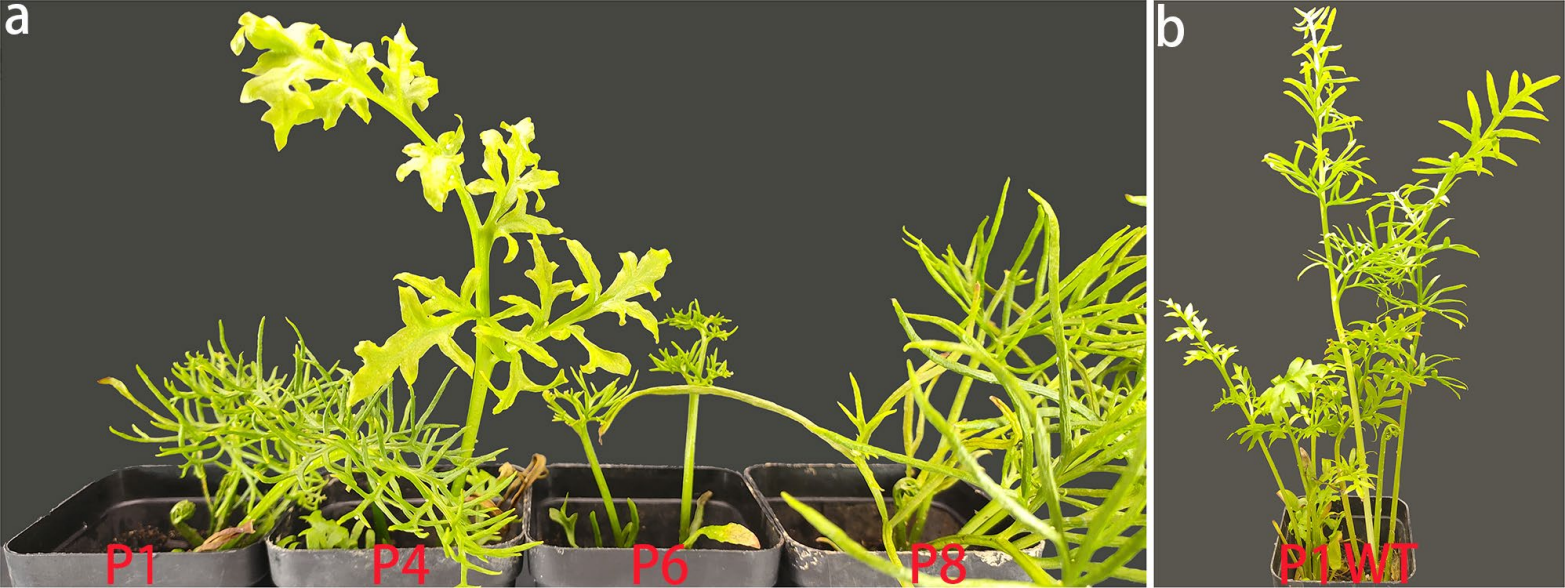
Morphologies of the edited plants. (**a**) Four T_0_ edited plants with the label of P1, P4, P6, and P8. (**b**) A T_1_ plant (P1) in comparison with a wild-type plant (WT)

Among the four T_0_ plants that were edited, P8 was the one the most like wild-type plants in terms of stature (Fig. 4a). However, fronds that emerged later were extremely small in P8, as was observed in P6. Compared to P6, P1 had somewhat long leaves, pinnae, and pinnules. P4 kept in vegetatively growing and thus was unable to form fronds that is characteristic of stripe-like pinnae/pinnules and sporangia. P1 was employed to produce T_1_ plants for phenotype validation because the two involved alleles were both null. After a two-month aseptic culture in liquid MS medium, P1 progenies had an average number of leaves of 5.57 (*n* = 26), while wild-type plants had developed an average number of leaves of 6.01 (*n* = 69), thus indicating a significant difference (*p* < 0.01, student *t*-test). Similarly, the mean fresh weight of P1 progenies was 0.03 g, significantly smaller than the 0.05 g of wild-type plants (*p* < 0.01). Nevertheless, the mean of the longest root was 2.14 cm in P1 progenies, which was significantly different from the 1.92 cm in the wild-type plants (*p* < 0.05). However, P1 progenies had an average number of leaves of 7.64 at the mature stage of vegetative growth (Fig. 4b), which was not significantly different from the wild-type plants’ average number of leaves of 7.56. At the same time, leaves in P1 progenies were apparently shorter and smaller (Fig. 4b).

### Spatiotemporal expression of *CrPINMa*

**Fig. 5:**
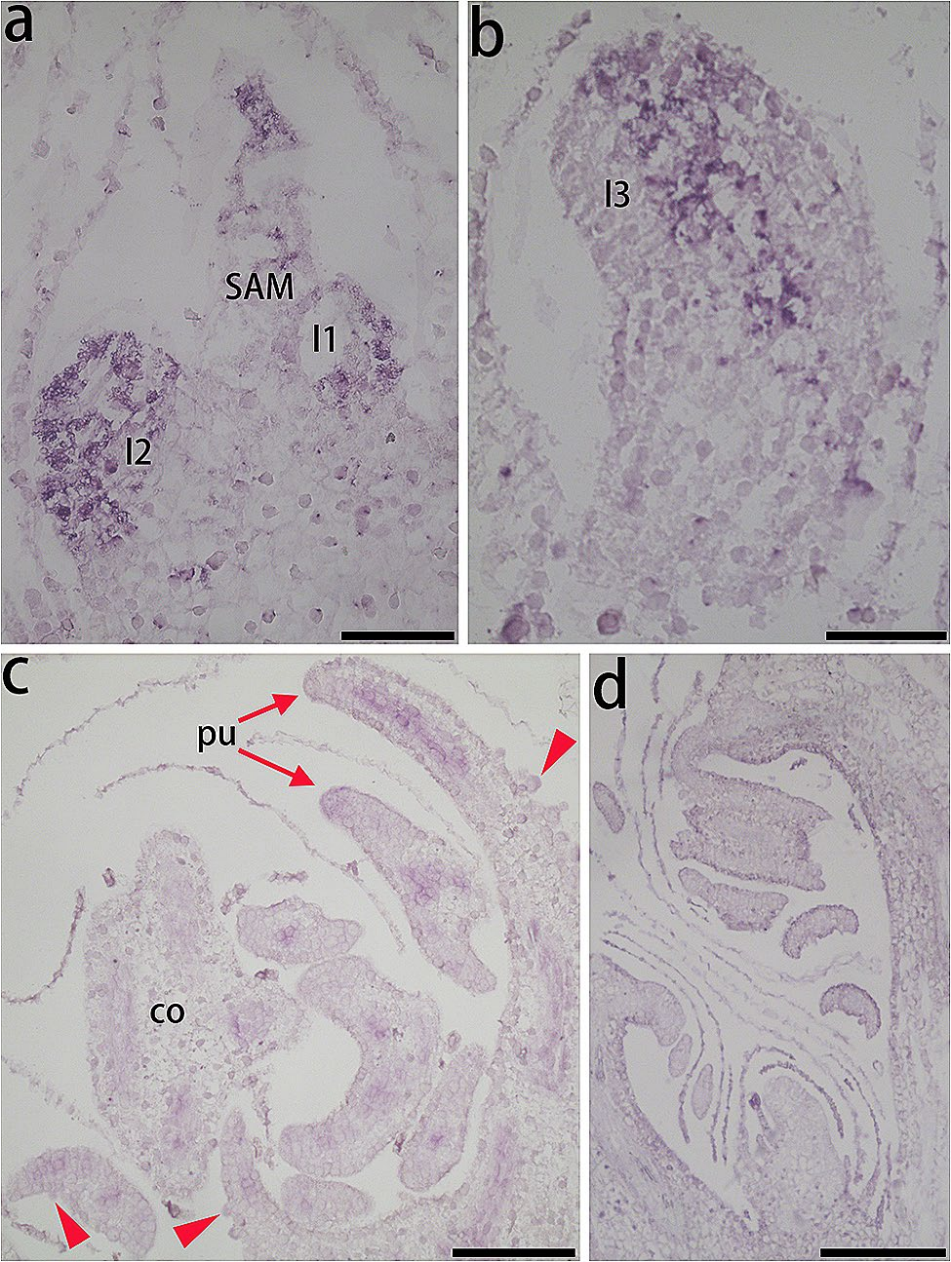
In situ hybridization of *CrPINMa*. (**a**) Expression in the SAM and leaf primordia. (**b**) Expression in the third leaf primordium. (**c**) Expression in fronds. (**d**) Sense probe hybridization. l1–l3, the first, second, and third leaf primordium. pu, pinnule. co, costa. Arrowheads indicated sporangia. Bar = 100 μm

*CrPINMa* was expressed in the shoot apical meristem, with a focus on the tetrahedral apical cell, which produces daughter cells to establish its underlying domain (Fig. 5a). Additionally, it was expressed in the epidermis of the first leaf primordium and throughout the second leaf primordium. In the third leaf primordium, *CrPINMa* expression was uneven, with the hybridization staining signal being specifically distributed along its central zone roughly corresponding to the putative vasculature (see Fig. 5b). This gene was weakly expressed in the costa vasculature while strongly in the pinnule vasculature, as observed in fronds characterized by sporangia and inward curled leaf margins (Fig. 5c). However, *CrPINMa* was indeed not expressed in sporangia. No staining signals were observed in sense probe hybridization (Fig. 5d).

### Subcellular localization of CrPINMa

**Fig. 6:**
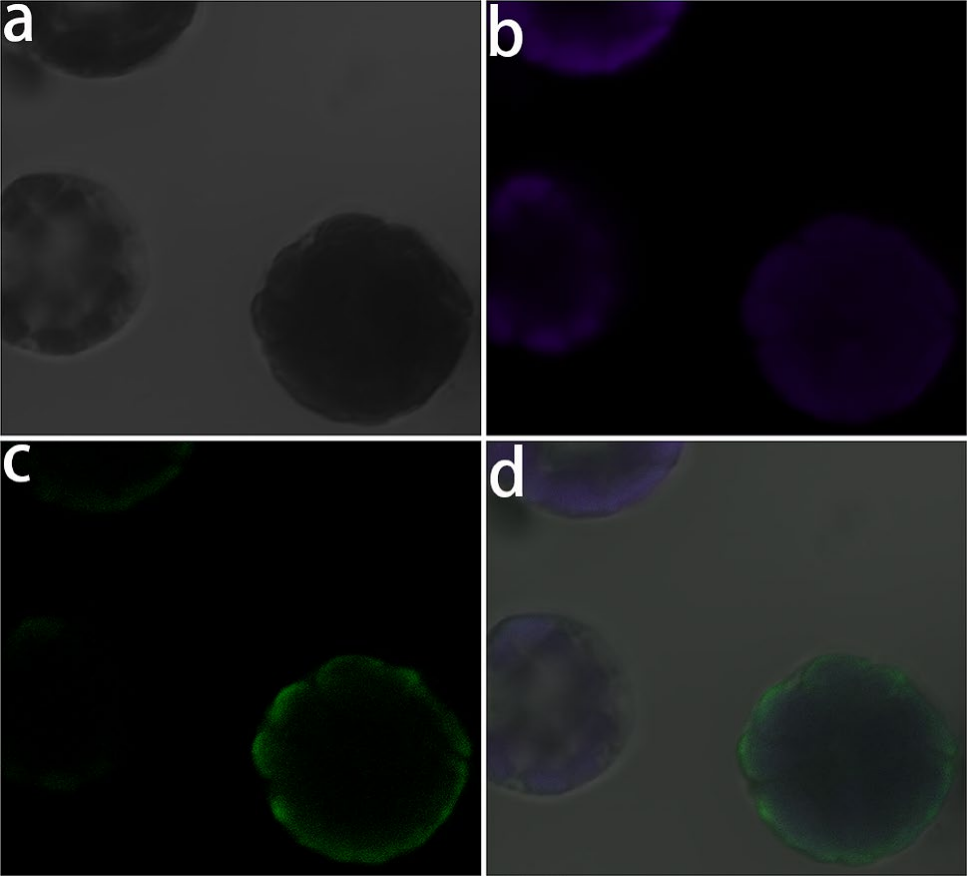
Subcellular localization of CrPINMa. (**a**) Protoplasts under differential interference contrast observation. (**b**) Fluorescence from chlorophyll. (**c**) Fluorescence from GFP. (**d**) The merged image

Among the protoplasts, some were completely dark, some had a dark periphery, and some displayed dark spots along the periphery (Fig. 6a). The darkness observed suggested the presence of thick or dense regions or parts. Therefore, these protoplasts might have been in different states. However, all protoplasts emitted chlorophyll fluorescence, usually from the places of those dark parts, thus indicating that they represented putative chloroplasts (see Fig. 6b). All protoplasts emitted the green fluorescent protein (GFP) fluorescence from a thin layer underneath the cell periphery, and it was weaker and more restrictedly distributed relative to the chlorophyll fluorescence (Fig. 6c, d).

## Discussion

### *CrPINMa* origination under gene duplication

Both *CrPINMa* and *CrPINMb* are from *Ceratopteris richardii* and correspondingly are tightly clustered together in the phylogenetic analysis. They both have equivalents in the same genus species *C*. *pteridoides* [32]. However, orthologs of *CrPINMa* cannot be found in the closely related *Adiantum capillus-veneris* or the relatively ancestral *Azolla filiculoides* and *Salvinia cucullata* [33–35]. Therefore, *CrPINMa* has undergone a gene duplication at the genus level. *CrPINMa* belongs to the PINM clade, and all genes within this clade are from species belonging to the core leptosporangiates. Furthermore, the PINK, PINL, and PINN clades also consist of genes from these species [36]. Therefore, *CrPINMa* is also associated with gene duplication above the genus level. *CrPINMa* is orthologous to angiosperm *PIN6* genes and even gymnosperm PINI genes. This finding is consistent with a previous study [36], indicating that *CrPINMa* belongs to a gene lineage distinct from the lineage that contains seed plant *PIN1*/*2*/*3*/*4*/*7* and their fern orthologues. Thus, *CrPINMa* is finally related to a gene duplication that occurred before the diversification of euphyllophytes, namely ferns and seed plants, with the formation of two independent lineages that can be traced back to the lycophyte genes and further to the bryophyte genes. *CrPINMa* may have displayed dosage effect, subfunctionalization, or neofunctionalization conferred by gene duplication [37], and furthermore it may have co-evolved with other duplicates [38]. Ultimately, changes in cis elements and protein residues may have made it unique to *C. richardii* [39].

### Practicability of CRISPR/Cas9 genome editing in ferns

CRISPR/Cas9 genome editing has not been previously reported in ferns, but it can occur in *C*. *richardii* here. Our practice is characteristic of the proximity of the two employed gRNA sites, which are just separated by a single nucleotide. Typically, two gRNA sites are distant from each other by 350–750 bp [30]. This close adjacency has efficiently produced genome editing in *CrPINMa*, meaning more unanticipated gRNA sites can be chosen in such experiments. Remarkably, each gRNA can bring about genome editing under this condition. Nevertheless, these two gRNA sites are oriented away from each other rather than facing each other, which may still guarantee a relatively large working space for the two units of the genome editing machine. The resulting mutations include both insertions and deletions, which have caused missing fragments and, unexpectedly, local frameshifts restricted to the gRNA-spanning region. It is possible that local frameshifts cannot occur when two gRNAs are located relatively far apart from each other [30]. The use of the fern *U3* promoter and terminator for gRNA expression, together with the fern *ACTIN* promoter for Cas9 driving, may also be positive in generating diverse mutated *CrPINMa*. Though there may be multiple vector insertions in different or the same chromosomes under bombardment manipulation, it is still possible to obtain edited plants free of foreign DNA as pure variations for *Ceratopteris richardii*, which is particularly important in CRISPR/Cas9 genome editing for food production and medical applications. The P1 edited individual is attractive because both *CrPINMa* alleles are loss-of-function in it, which is useful for implicating gene function. In addition, the mutations remain at the transcriptional level, and unexpected damage is added. Therefore, *CrPINMa* is genetically and functionally null in the P1 line, as there is no interference from alternative transcripts that would have bypassed the mutated site.

### Role of *CrPINMa* in leaf development

Disrupting *CrPINMa* still allows T_0_ plants to survive and produce fronds with sporangia, which indicates for this gene a subtle role in development in *C*. *richardii*. However, fronds, pinnae, and pinnules may become miniature in these T_0_ plants. In the T_1_ generation, spores can germinate to generate gametophytes, followed by fertilization and sporophyte formation. However, young sporophytes have fewer leaves, and at the same time fresh weight is reduced though it is taken for granted that leaves are the main component of these plants. As a proof, roots are somewhat longer at the same time, possibly due to growing with a lower density. Furthermore, both homozygotes and heterozygotes of P1a and P1b have displayed these three phenotypes. However, when these sporophytes reach the apex of vegetative growth, they no longer differ in leaf number. Thus, *CrPINMa* disruption can convincingly slow down leaf initiation. At the same time, these fully developed leaves are always short and small, which is consistent with the formation of the miniature fronds in T_0_ plants, collectively indicating that *CrPINMa* disruption can also reduce leaf size.

*CrPINMa* is not in the same lineage with *PIN1*, but leaf initiation can also be inhibited in the *pin1* mutant, which is characterized by a long and variable plastochron [19]. Unfortunately, there is no assay of leaf size in the *pin1* mutant [19]. Instead, *CrPINMa* is in the same lineage with *PIN6*. However, *PIN6* lacks some conserved motifs characteristic of the long but not the short central hydrophilic loop, thus being treated as the non-canonical type while *CrPINMa* is the canonical type. Moreover, *PIN6* is involved in root formation [40], in leaf venation [41], and so on, with localization to either the PM or the ER. *Cardamine hirsute* has compound leaves, and its equivalent of *PIN1* is necessary for the formation of leaflets [17]. Furthermore, disrupting the *PIN1* ortholog *SMOOTH LEAF MARGIN1* (*SLM1*) in *Medicago truncatula* results in an increase in the number of terminal leaflets but a decrease in that of lateral leaflets [42]. Finally, compound leaf formation throughout land plants has been found concerning the independent recruitment of Class I *KNOTTED-LIKE1 HOMEOBOX* (*KNOX*) genes [43]. Thus, *CrPINMa* may be not the crucial gene responsible for the compound leaves in *C*. *richardii*. Though CRISPR/Cas9 genome editing is concerned for off-target effect, different *CrPINMa* disruptions have concomitantly produced the same phenotype.

### Tempospatial expression and subcellular localization of *CrPINMa*

*CrPINMa* is expressed in the SAM, leaf primordia, and leaf vasculature, which is consistent with the fact that *CrPINMa* disruption has resulted in retarded leaf emergence and reduced leaf size. Similarly, *Arabidopsis PIN1* is expressed in the SAM [44], leaf primordia [14, 15, 45], and leaf vasculature [46, 47], and meanwhile determines time of leaf initiation. In contrast, other four *Arabidopsis PIN*s that also possess a long hydrophilic loop are only expressed in the epidermis of leaf primordia, thus exerting no obvious influence on leaf development [19, 41]. *PIN5*/*6*/*8* possess short hydrophilic loops, but they can be expressed in leaf vasculature to play a role in venation formation in corporation with *PIN1* [41, 48, 49]. Therefore, it is possible that *CrPINMa* can specify leaf vasculature without an intracellular partner, as there is no PINs possessing a short hydrophilic loop in *C*. *richardii*. It is bold to take *PIN-LIKES* (*PILS*) that can contribute to intracellular auxin transport into consideration for vasculature formation in vascular plants [50].

GFP is not a membrane protein but is localized to the PM after being fused with CrPINMa, indicating that CrPINMa tends to be distributed in the PM. PINs, which typically have a long hydrophilic central loop and characteristic motifs within it [36], are generally localized to the PM and further to its specific cell side [10]. Consequently, auxin flow directionality can set up organ positions before they ultimately form during plant morphogenesis [14, 44]. The localization of CrPINMa to the PM can be supported by its sequence characteristics, and therefore it is likely distributionally polarized in the PM too. Thus, disrupting *CrPINMa* may have disturbed auxin maxima putatively important to the initiation of leaf primordia and pinna/pinnule primordia in *C*. *richardii*, thus delaying leaf emergence and reducing leaf size.

## Conclusions

*CrPINMa* belongs to the canonical type and there is not non-canonical PINs lacking a long central hydrophilic loop and its characteristic motifs. *CrPINMa* has evolved under various levels of gene duplication, which means it may be specific to the corresponding species though in the same lineage with *Arabidopsis PIN6* but not *PIN1*. The CRISPR/Cas9 genome editing technique is practicable in *C*. *richardii* and can produce diverse mutations when design is optimized. *CrPINMa* plays a role in leaf emergence and leaf outgowth. Consistently, *CrPINMa* is expressed in the SAM, leaf primordia, and leaf vasculature, and its protein products are localized to the PM. In summary, *CrPINMa* may have functioned in fern development like classical *PIN*s.

## Methods

### Materials

The water fern strain *Ceratopteris richardii* Hnn, gifted by Andrew Plackett from Jane Langdale’s lab, was used. Spores were first imbibed under darkness for two days, and then sterilized using a 1∶5 diluted commercial bleach for 30 min under rocking, and finally washed five times using autoclaved water, for a germination in 20 mL of liquid Murashige and Skoog (MS) medium containing 2% sucrose using 9-cm diameter petri dishes. Germination, fertilization, and early growth until the formation of 6‒7 leaves were performed in the culturing chamber under 25 ℃ and 16-h illumination. Callus was induced from aseptic sporophytes that had developed 3‒4 leaves using the solid MS medium supplemented with 5 µM 6-benzylaminopurine (BA) [51]. Callus was sub-cultured every 2 weeks and this procedure was performed less than three times before bombardment transformation.

### Phylogenetic tree construction

Protein sequences of *PIN*s were collected from previous research [36] and FernBase (https://fernbase.org/), with a focus on clarifying *CrPINMa* phylogeny. Sequences were aligned using Clustal Omega (https://www.ebi.ac.uk/Tools/msa/clustalo/). The N- and the C-terminus of PINs were conserved, and they were chosen for the formation of a multi-alignment consisting of 377 amino acids in a smaller dataset and a multi-alignment consisting of 312 amino acids in a larger dataset. The maximum-likelihood method was used and FastTree (http://meta.microbesonline.org/fasttree/) was called to generate phylogenetic trees, and trees were visualized using MEGA X (https://www.megasoftware.net/dload_win_gui). Protein sequence region between the two conserved terminal domains roughly corresponded to the so-called central hydrophilic loop useful in distinguishing canonical and non-canonical *PIN*s, and this region was screened for motifs using MEME (https://meme-suite.org/meme/tools/meme). Gene accessions were provided (see Table S1).

### Genetic transformation

The promoter (chromosome 1, 46482269.46481782) and the terminator (46481579.46481220) of a *U3* gene were determined with the help of the draft genome for *C*. *richardii* [52]. Similarly, the promoter (chromosome 8, 77831923.77830277) of an *ACTIN* gene, specifically 08G028400, was identified. The pGTR and RGEB32 plasmids were obtained from Addgene (https://www.addgene.org/). The *U3* components and the gRNA scaffold from RGEB32 were linked together using overlapping polymerase chain reaction (PCR). The resulting product was then digested using HindIII/BstBI for replacement of the original rice *U3* promoter and terminator. The *ACTIN* promoter substituted for the original rice *UBIQUITIN* promoter for Cas9 driving, with the aid of KpnI/BstBI cutting and in-fusion ligation (https://www.takarabiomed.com.cn/). Therefore, a CRISPR/Cas9 genome editing vector potentially preferred by ferns was created. The vector, named pFGEB (see Fig. S3), could be cut with BsaI and ligated to tRNA-gRNA structures treated with FokI, as in the original protocol [30]. The online Broad Institute (https://portal.broadinstitue.org/gpp/public/analysis-tools/sgrna-design) was visited for gRNA binding site screening. Primers were available (see Table S2).

A combination of 50 µL gold powder and 5 µL plasmid DNA (approximately 0.5 µg) was vortexed for 1 min, and then 50 µL of 2.5 M CaCl_2_ and 20 µL of 0.1 M spermidine were successively added into. This mixture was centrifugated under 5000× g for 1 min, and the precipitate was resuspended with 150 µL 75% alcohol. The suspension was similarly centrifugated and then was resuspended with 150 µL 100% alcohol. After the third centrifugation, the precipitate was suspended with 20 µL of 100% alcohol, getting ready for two bombardments. A GJ-1000 gun (Xinzhi Ltd, Ningbo, China) was employed, and ca. 7 MPa N_2_ stream was generated, for a ca. 1 cm flying of the vector film before it hit against the stopping screen, which would generate particles piercing into callus cells ca. 11 cm away.

Before bombardment, callus was cultured in the solid MS medium containing 5 µM kinetin (KT) for 4 days [51]. After bombardment, callus was maintained on the original KT-containing medium for additional 3 days. Subsequently, callus was selected on the KT-containing medium with the addition of 50 mg/mL hygromycin for 2 weeks. Immediately, callus was selected on the medium only with the addition of hygromycin for 2 weeks, which was repeated three times, meaning a process of a total of one month approximately. Finally, the regenerated plantlets were planted in cubes that contained potting mixtures, and spores were harvested from yellowed, aged fronds.

Cetyltrimethylammonium bromide (CTAB) was used in isolating genomic DNA, and then purified PCR products were sequenced using Sanger method with one of amplification primers (see Table S2), by Sangon (Shanghai, China). In reference to original sequence, edited sequences were identified from chromatogram files. To validate edited sequences, bacterial transformation was conducted and then clones were separately sequenced.

### RT (reverse transcription)-PCR analysis

Total RNA was extracted from a handful of young P1 progeny having 5‒6 leaves for each sample using the MiniBEST Plant RNA Extraction Kit (TAKARA China, Beijing, China), and reverse transcription was performed using PrimeScript RTase (TAKARA) and the poly(T) primer appended with an adaptor. Two pairs of primers, located separately at the 5’ and 3’ untranslated region (UTR), were used in a nested PCR to amplify the full-length cDNA for *CrPINMa*. The products were directly sequenced using a sequencing primer focused on the mutated site and another primer focused on two exon-intron junctions. Primers were available (see Table S2).

### In situ hybridization

Plant tissues were fixed in FAA for approximately 12 h. They were then dehydrated, cleared, embedded, and sectioned with 8 μm thickness for an anchoring on poly-L-lysine-coated glass slides. The whole coding region of *CrPINMa*, ranging from the start codon to the stop codon, was cloned into the T-easy vector (Promega China, Beijing, China). PCR was performed using a gene-specific forward primer and a reverse primer located upstream of the T7/SP6 promoter to generate templates for RNA probe synthesis. Probes, labeled with digoxigenin, were hydrolyzed, and denatured before being hybridized with target transcripts in sections that had been treated with 2 µg mL^− 1^ proteinase K under 37 ℃ for 30 min. Following probe hybridization, slides were washed with 0.2× SSC under 55 ℃ for 1 h, and this washing step was repeated once. After prebinding, antibodies were bound at room temperature for 2 h. Subsequently, they were stripped off by four washings, taking a total of 1 h. Alkaline phosphatase was used to color tissues if containing target transcripts.

### Localization using the GFP translational fusion protein

The pCAMBIA-1309 binary vector containing the *MGFP5* gene was digested with NcoI and BglII enzymes. The *CrPINMa* full-length coding sequence was acquired using primers compatible with in-fusion technology. They were ligated together, with the *MGFP5* gene located at the C-terminus and losing its first two codons, whereas the preceding *CrPINMa* losing its stop codon. The *35 S: CrPINMa-MGFP5* construct was then used to transform *Arabidopsis* leaf protoplasts. Observation was carried out using a laser scanning confocal microscope, specifically the A1 + Ti2 (Nikon, Tokyo, Japan).

### Electronic supplementary material

Below is the link to the electronic supplementary material.

Supplementary Material 1

Supplementary Material 2

Supplementary Material 3

Supplementary Material 4

Supplementary Material 5

Supplementary Material 6

Supplementary Material 7

Supplementary Material 8

## Data Availability

All data generated or analysed during this study are included in this published article [and its supplementary information files].
