# Supplementary figures and images for: Control of leaf development in the water fern Ceratopteris richardii by the auxin efflux transporter CrPINMa in the CRISPR/Cas9 analysis
Source: BMC Plant Biol. 2024 Apr 24;24:322. doi: 10.1186/s12870-024-05009-4 (PMC11040788; doi:10.1186/s12870-024-05009-4)

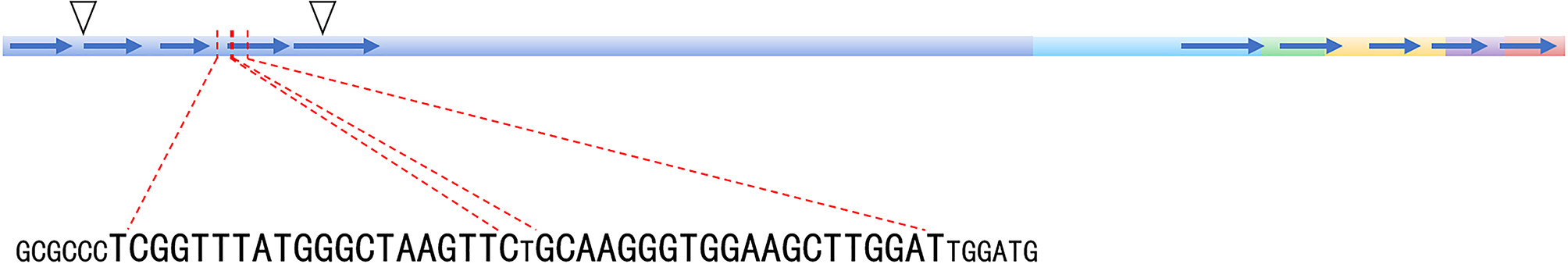

Supplement: Supplementary file 1 — Supplementary Material 1 [file 12870_2024_5009_MOESM1_ESM.tif]

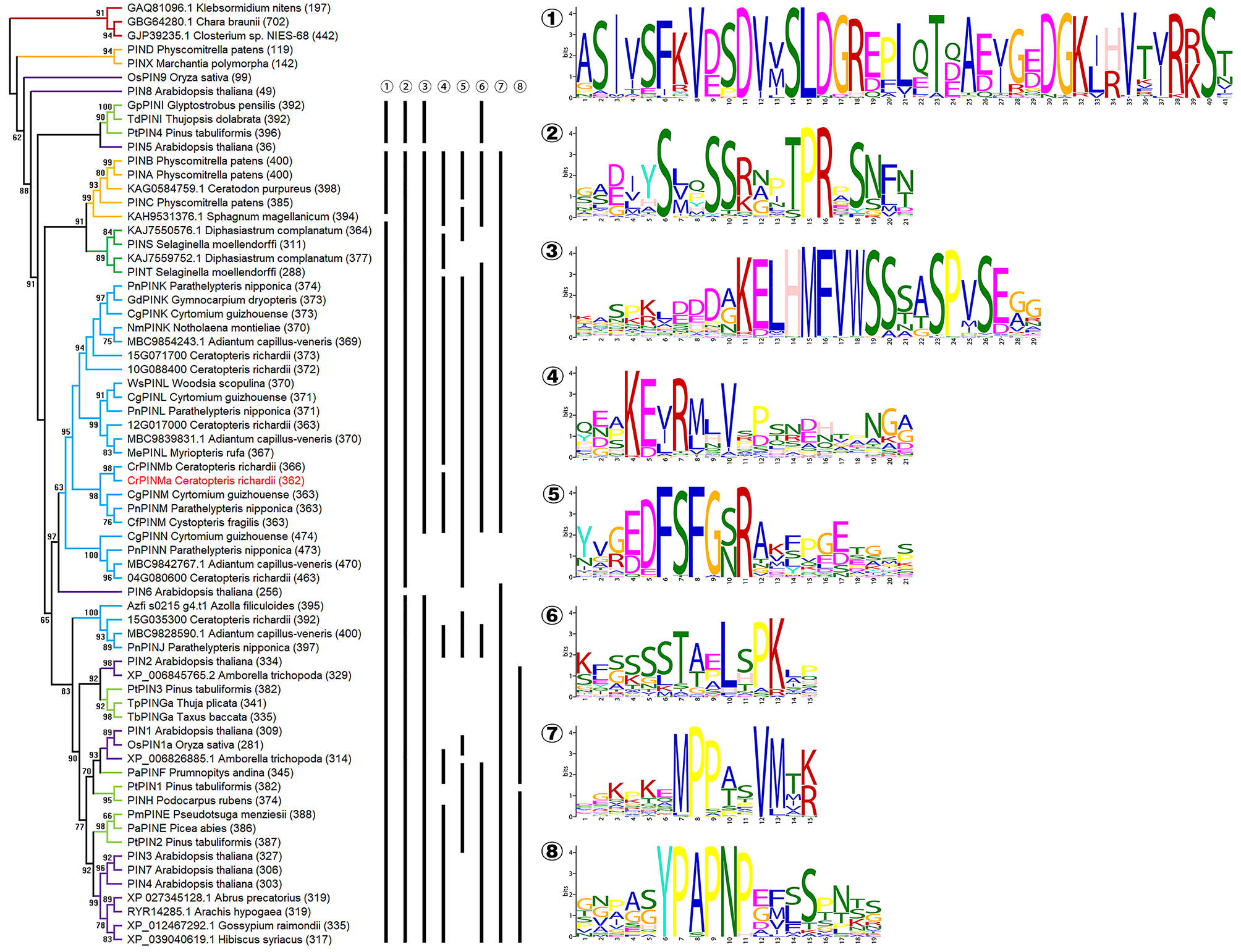

Supplement: Supplementary file 2 — Supplementary Material 2 [file 12870_2024_5009_MOESM2_ESM.tif]

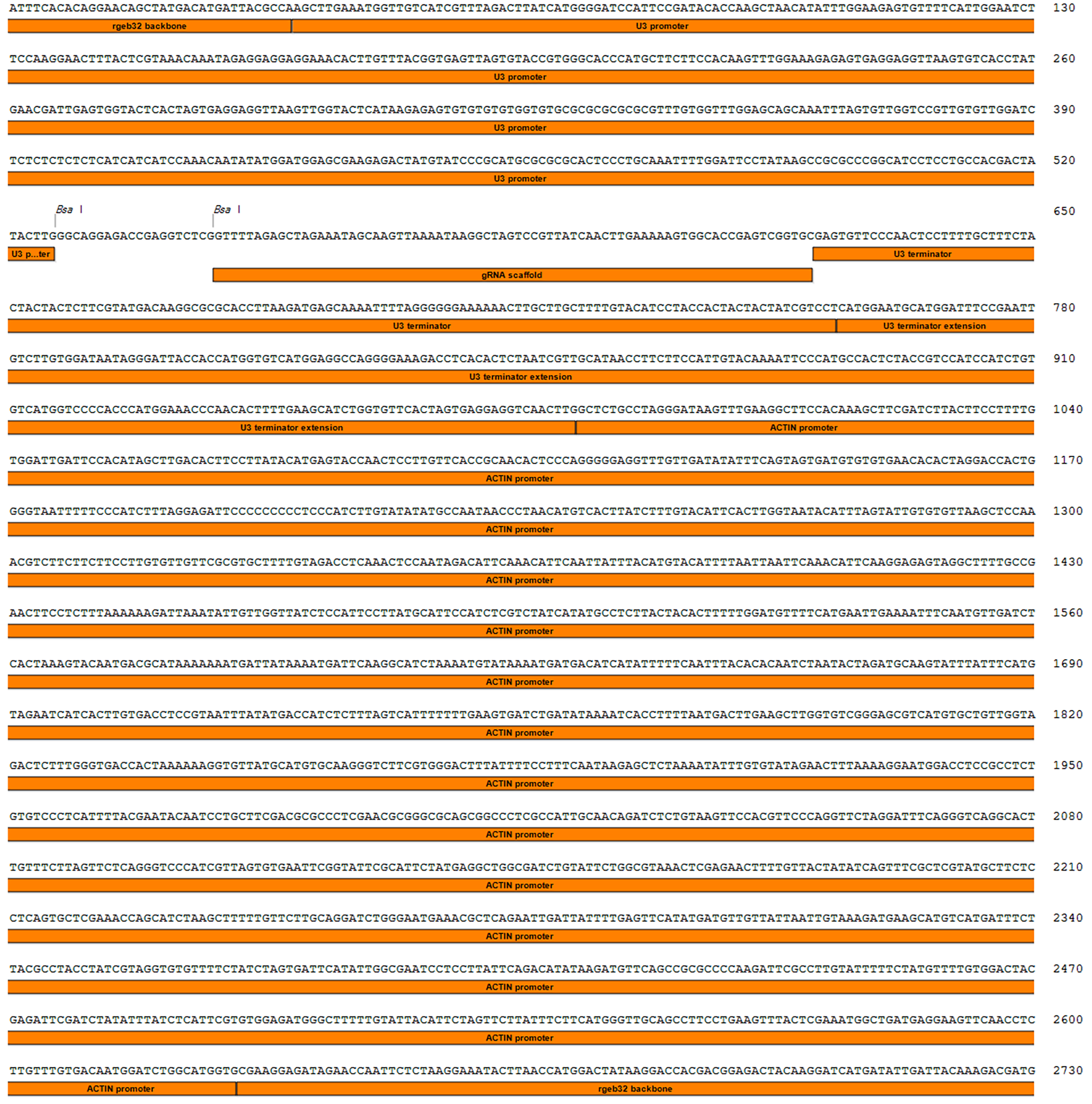

Supplement: Supplementary file 4 — Supplementary Material 4 [file 12870_2024_5009_MOESM4_ESM.tif]

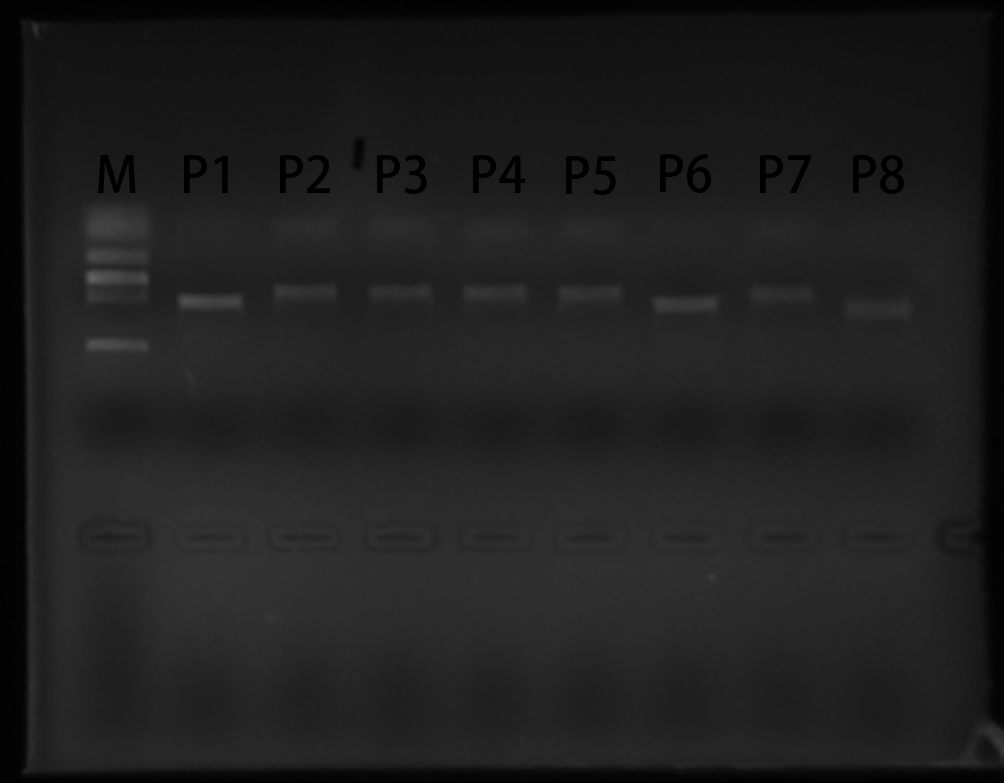

Supplement: Supplementary file 5 — Supplementary Material 5 [file 12870_2024_5009_MOESM5_ESM.tif]

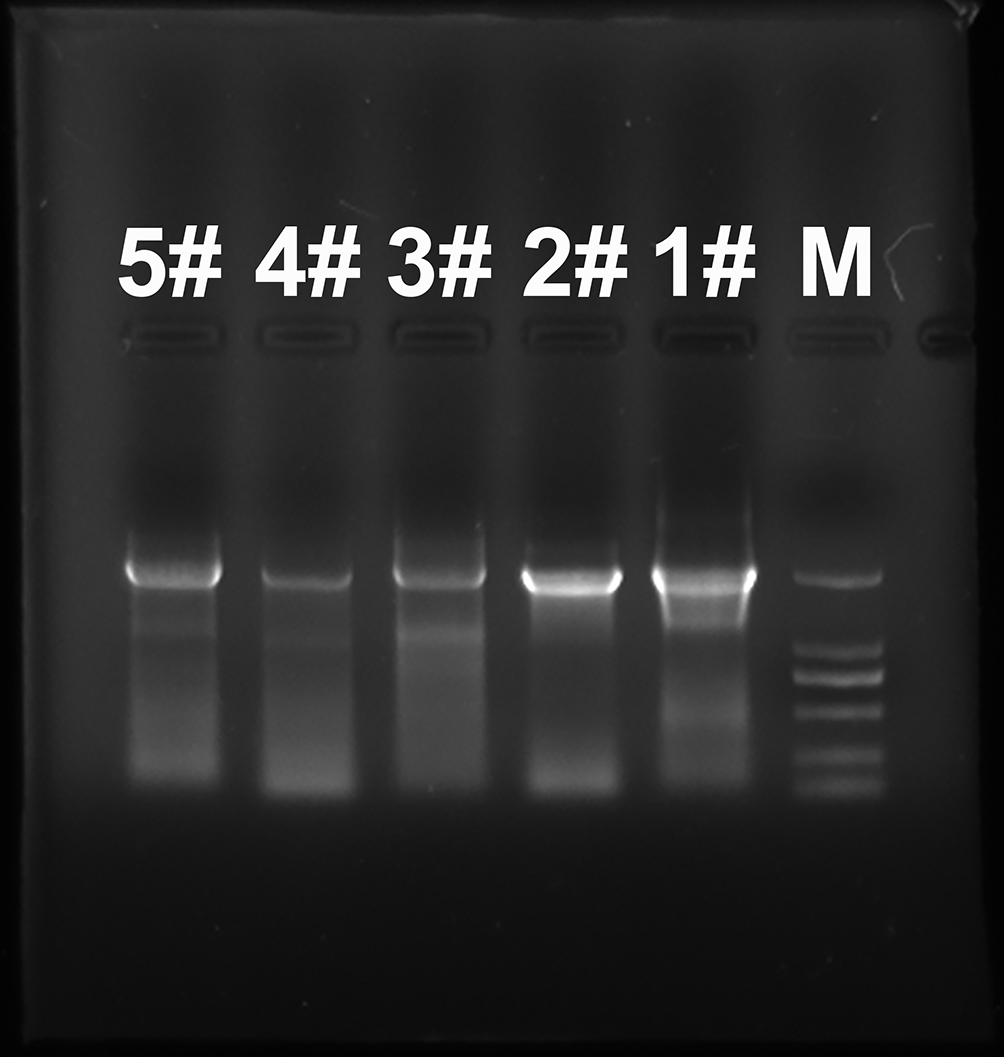

Supplement: Supplementary file 6 — Supplementary Material 6 [file 12870_2024_5009_MOESM6_ESM.tif]
