# Supplementary material for: Control of leaf development in the water fern Ceratopteris richardii by the auxin efflux transporter CrPINMa in the CRISPR/Cas9 analysis
Source: BMC Plant Biol. 2024 Apr 24;24:322. doi: 10.1186/s12870-024-05009-4 (PMC11040788; doi:10.1186/s12870-024-05009-4)
Supplement: Supplementary file 7 — Supplementary Material 7 [file 12870_2024_5009_MOESM7_ESM.doc]

Table S2 Primers in this study

| Primer names | Prime sequences |
| --- | --- |
| For U3 promoter cloning |  |
| U3F497 | CAAGGTTTTGCGTGAGGGAA |
| U3-2BsaI | ccgagacctcggtctcctgccCAAGTATAGTCGTGGCAGGAG |
| For U3 terminator cloning |  |
| U3-F | GAGTGTTCCCAACTCCTTTTG |
| U3R1991 | GCCTCCCTTCAAGAAAATGGA |
| For sgRNA cloning |  |
| 2BsaI | ggcaggagaccgaggtctcgg |
| gRNA-U3-R | CAAAAGGAGTTGGGAACACTCgcaccgactcggtgccacttt |
| For actin promoter cloning |  |
| pACT7998F | GAGGAGGTCAACTTGGCTCTGCCTAGGGATAAGTTTG |
| pACT9625R | GTTCTATCTCCTTCGCACCATGCCAGATCCATTGTCAC |
| For gene-editing detection |  |
| C1a102F | CACTCCAGTTCAATGCTCGG |
| C1R1119 | GGTTTTATCTCGGCGCCCTTTCTA |
| For transcript amplification | |
| flPINM-f65 | TTCCTCATCTGAAGTAGTCTTAGCC |
| flPINM-f72 | TCTGAAGTAGTCTTAGCCAGCGAAT |
| flPINM-r2196 | AAAAACGATCATTTGGTCCTCATAA |
| flPINM-r2236 | TCTTATGTAATATGCCCAAAATGTG |
| For transcript sequencing | |
| C1a102F | CACTCCAGTTCAATGCTCGG |
| PINM-f53 | TACGGATGGTTGGCCGAAAG |
