# Supplementary material for: Control of leaf development in the water fern Ceratopteris richardii by the auxin efflux transporter CrPINMa in the CRISPR/Cas9 analysis
Source: BMC Plant Biol. 2024 Apr 24;24:322. doi: 10.1186/s12870-024-05009-4 (PMC11040788; doi:10.1186/s12870-024-05009-4)
Supplement: Supplementary file 8 — Supplementary Material 8 [file 12870_2024_5009_MOESM8_ESM.doc]

**Supplementary Information**

Fig. S1: .tif, A scheme of the *CrPINMa* coding sequence. Rectangles filled with different colors indicated exons, and the superimposed blue arrows indicated transmembrane regions. Red dashes indicated positions and sequences of the two gRNA sites (namely gRNA257 and gRNA278), and triangles indicated positions of primers used in detecting gRNA-induced mutations

Fig. S2: .tif, Phylogenetic analysis of *CrPINMa*. *CrPINMa* was evaluated in a larger tree (left) and investigated in motif presence indicated by bar covering (middle) and motif types indicated by sequence logo (right). Numbers of amino acids within central loops were indicated in brackets following gene names. ①–⑧ motifs had been identified

Fig. S3: .tif, The pFGEB map

Fig. S4: .tif, The uncropped gel in Fig. 2

Fig. S5: .tif, The uncropped gel in Fig. 3

Table S1: .xlsx, Accession numbers of genes

Table S2: .doc, Primers employed in this study
